# Local Immunoglobulin E in nasal polyps: Role and modulation

**DOI:** 10.3389/fimmu.2022.961503

**Published:** 2022-09-08

**Authors:** Yang Shen, Nan Zhang, Yucheng Yang, Suling Hong, Claus Bachert

**Affiliations:** ^1^ Department of Otorhinolaryngology, The First Affiliated Hospital of Chongqing Medical University, Chongqing, China; ^2^ Upper Airways Research Laboratory, Department of Otorhinolaryngology, Ghent University, Ghent, Belgium; ^3^ Division of Otorhinolaryngology Diseases, Department of Clinical Sciences, Intervention and Technology (CLINTEC), Karolinska Institute, Stockholm, Sweden

**Keywords:** Immunoglobulin E, local B cell, nasal polyposis, Type2 inflammation, *Staphylococcus aureus*

## Abstract

In the airway, IgE is traditionally regarded as a key mediator in allergic diseases, such as AR and allergic asthma. However, growing evidence demonstrates the importance of local IgE in airway inflammatory diseases, irrespective of the presence of allergy. In this review, we discuss the most recent evidence for IgE in chronic rhinosinusitis with nasal polyps(CRSwNP), including the local IgE’s characteristics, the modulation of its synthesis, and its function. The levels of local IgE are significantly elevated in polyps independently of IgE serum levels and atopic status. Local IgE, which is correlated with type 2 inflammation, is polyclonal and functional. IgE is produced by active B cells and is dependent on the class switch recombination(CSR). In NPs, this process is triggered by not only allergens but also microbial colonization, especially the superantigen- *Staphylococcus aureus*. The production of local IgE is modulated by lymphocytes(such as Tfh, ILC2s, iTreg), cytokines(such as IL-4, IL-13, IFN-γ, TGF-β, IL-2, IL-21), transcription factors, and B cell-intrinsic factor. Due to the central role of IgE in NPs, it is regarded as an ideal target for therapy and has been proved to be clinically successful. Based on this knowledge, we believe that exploring the trigger and regulatory factors for the activation of local B cells and CSR to IgE will provide more valuable information for us to recognize the pathological mechanisms of local IgE and offer the possible option for new therapeutic targets of nasal polyps.

## Introduction

Immunoglobulin E(IgE) has an essential function in immunity to parasites and type I hypersensitivity. Although IgE is typically the least abundant isotype, it is one of the responsible antibodies for the classical adaptive immune response and is capable of triggering the most powerful inflammatory reactions after binding to its specific receptors (FcϵRI, the high-affinity IgE receptor; FcϵRII, also known as CD23, the low-affinity IgE receptor).

Traditionally, IgE is regarded as a key mediator in the pathophysiology of allergic diseases, such as allergic rhinitis(AR), allergic asthma, anaphylaxis, and food allergy. People with a tendency to develop allergic reactions when exposed to antigens initially produce antigen-specific IgE during sensitization. Subsequent exposure to its specific sensitizing allergen, IgE mediates antigen cross-linking of the high-affinity IgE receptor (FcϵRI) on a variety of cells (e.g. mast cells, basophils, dendritic cells, monocytes, and smooth muscle cells). Release of mediators by mast cells and basophils follows, triggering classic symptoms of allergy (nasal congestion, wheezing, sneezing, whealing, etc.)

However, our concept of the role of IgE in disease states is undergoing reassessment and re-evaluation., Growing evidence shows that IgE also plays a role in non-allergic airway diseases. Increased local IgE production was found in nasal polyps(NPs) regardless of systemic atopic status (1–4). Class switch recombination(CSR) to IgE was reported in the bronchial mucosa of both atopic and nonatopic patients with asthma (5). In addition, our clinical trials showed that omalizumab was effective in CRSwNP patients with or without asthma. independent of results of allergy tests (6). All these studies indicate the importance of local IgE in the airway, irrespective of the presence of allergy. Exploring the biological functions of IgE in these “non-allergic” diseases, therefore, is meaningful in terms of therapeutic interventions.

Even if IgE is involved in both allergic and nonallergic airway diseases, its role may vary in these diseases. In AR, IgE concentrations in serum and mucosal tissue homogenates are highly correlated, while they fail to show relevant correlations in chronic rhinosinusitis with nasal polyps(CRSwNP) and comorbid asthma (7). Although in both cases allergen-specific, IgE in AR and allergic asthma is often oligoclonal or monoclonal, while IgE in CRSwNP, often comorbid late-onset non-allergic asthma, is predominantly polyclonal (7). Furthermore, the formation of germinal center(GC)-like structures in NPs, has not been reported in the nasal mucosa of AR (8–10).

In this review, we focused on the role of IgE in NPs. We summarized the characteristics of local IgE in NPs, then analyzed the possible pathway of local IgE synthesis and its modulation, and finally discussed the function of IgE in NPs, including recent information from targeting IgE.

## The characteristics of IgE in NPs

### IgE is significantly increased

In NPs, tissue IgE appears to be increased in patients from all regions (11). The concentrations of total IgE are significantly increased in mucosal tissue compared with those in serum and they were also significantly elevated compared with those in non-NP tissue (7, 12, 13); this has been reported in both European and Asian populations (8, 14, 15). In addition, the IgE concentrations in mucosal tissue homogenates showed no correlation with those in serum, implying the possibility of local IgE production rather than the tissue IgE representing overspill from the peripheral blood. Moreover, IgE-positive plasma cells, IgE-positive B cells, secondary lymphoid tissue, and GC-like structures were found in polyp tissue, adding another argument for the local production of IgE in NPs (8, 10, 16, 17). Further evidence for local IgE synthesis within the polyp tissue has come from studies of cultured B cells derived from NPs secreting high levels of IgE antibodies *in vitro (* 18). Interestingly, local hyper-immunoglobulinaemia E was present in both atopic and nonatopic patients (1, 2, 12) and unrelated to skin test results (3, 4). These data suggest that continuous local IgE production occurs, which is derived from many different B cell clones, even in the absence of a specific allergen stimulus or in the presence of a so far unknown allergen, to maintain IgE levels.

### Local IgE is polyclonal and functional

IgE antibody in CRSwNP appear to be polyclonal and covers a broad spectrum of allergens/antigens rather than oligoclonal ones like in AR (7, 19). Local IgE in the nasal polyp mucosa is pro-inflammatory. The ability of polyclonal IgE to activate mast cells in polyps was first proposed after observations in tissue from patients with NP vs. AR. Then, mucosal IgE antibody in NP was found to induce mast cell degranulation in response to numerous inhalant allergens (7). Meanwhile, polyclonal IgE in polyp homogenates promoted the CD23-mediated pro-allergic responses and induced basophil activation and histamine release (19). Thus, distinguishing from the oligoclonal IgE in AR, which only reacts to specific antigens, the IgE in NPs is polyclonal, which reacts to multiple antigens and derives from many B cells; Then, the polyclonal IgE can initiate mediators releasing *via* interactions with FcϵRI on mast cells or FcϵRII(CD23) on basophil, finally contributing to the persistent local inflammation in CRSwNP.

### Local IgE is involved in T2 inflammation

The Type2(T2) inflammation in NPs includes higher levels of local IgE, IL-5 (20), IL-4, IL-25, IL-33 (21), thymic stromal lymphopoietin(TSLP) (22), eotaxin-2, monocyte chemoattractant protein(MCP)-4 (23), and tissue eosinophilia. A T2 inflammation is typical in European CRSwNP, while less prevalent in patients from Asia (11, 24). In recent years, increasing T2-dominated profiles have been observed in Asian CRSwNP (25). The increased local IgE in nasal polyp tissue is correlated with increased Eosinophil cationic protein (ECP), IL-5, eosinophilic infiltration, and the presence of staphylococcal enterotoxin(SE)-IgE and asthma comorbidity (11, 12), implicating the role of IgE in the mediation of T2 inflammation. In the cluster analysis of CRS patients based on immunological biomarkers, the high IL-5 clusters showed increased levels of total IgE and SE-IgE in nasal tissues and also a higher frequency of comorbid asthma (26). Whereas 30-50% of CRSwNPs in Europe are associated with asthma, 57% of SE IgE-positive polyps and 64%-71% of polyps with high IgE levels show significantly higher comorbidity rates with asthma (27, 28).

## The production of IgE in NPs

### Local B cells are active in NPs

**Figure 1:**
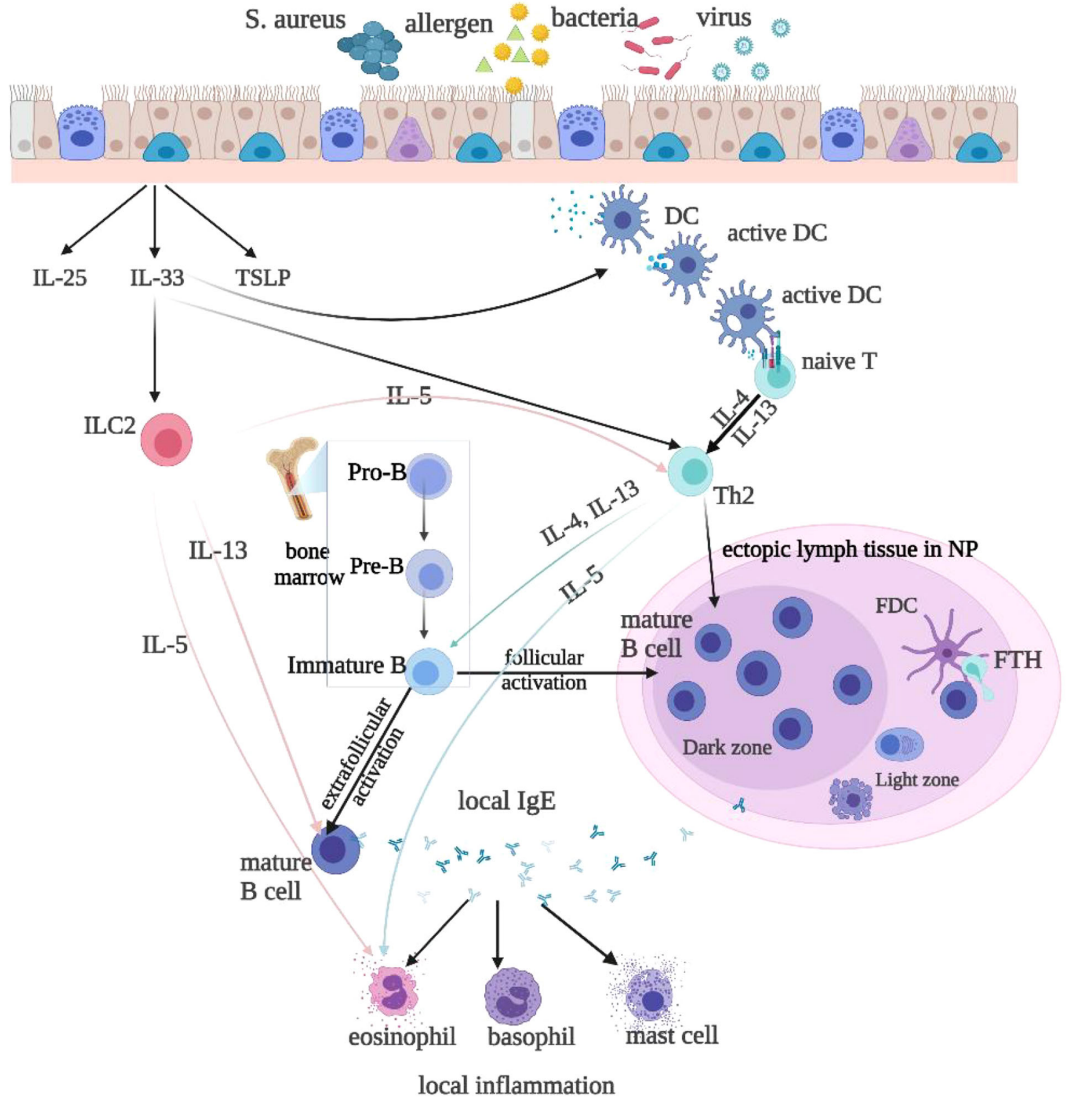
Local IgE is produced by follicular and extrafollicular activated B cells through class switch recombination. Microbial colonization, such as bacteria, rhinovirus, and allergens, especially the superantigen Staphylococcus aureus(SA), stimulate local IgE formation. The T follicular helper(Tfh) cells and Th2-like cells are helper cells for the B cell class switching to IgE. The Th2 cytokines IL-4 and IL-13 stimulate class switching to IgE and IgE formation. Type 2 innate lymphoid cells(ILC2s) produce Th2 cytokines, such as IL-4, IL-5, and IL-13, to promote type 2 inflammation, upon stimulation with epithelium-derived cytokines, such as IL-25, IL-33, and thymic stromal lymphopoietin(TSLP). IL-33 also activates DCs, which may *via* superantigens prime naive T cells to develop into Th2 cells. IL-4 and IL-13 then activate B-cells and initiate local IgE production. IL-5 released by ILC2s or Th2 cells activate eosinophils.

B cells differentiate into plasma cells and produce antibodies, so the differentiation and function of B cells in polyps directly influences the levels of local antibodies. In recent decades, the role of B cells and plasma cells has gained interest in CRSwNP(**Figure 1**). Early immunohistochemical and histopathologic research demonstrated that elevated expression of CD20 (naive B cell marker) and CD138 (plasma cell marker) were found in CRSwNP tissue vs inferior turbinate controls (8, 29), which were confirmed by a later flow cytometry study (30) and an immunofluorescence study (16). A recent report by Miljkovic D et al. demonstrated increased frequencies of naive and effector B–cell subtypes in the mucosa of patients with CRSwNP (31). Feldman S et al. found that CD19^+^CD27^+^CD38^hi^ plasmablasts were dramatically increased in CRSwNPs, which also had a higher frequency of expression of Epstein Barr Virus-induced protein 2(EBI2), compared to tonsils samples (18). Furthermore, this report showed that nasal polyp-derived B cells secreted high levels of antibodies *in vitro* compared to tonsil-derived B cells, including IgG, IgA, and IgE antibodies, confirming the activation of B cells from NPs (32). This study was supported by elevated levels of all antibody isotypes in NPs, except IgD (13, 16, 30). These studies indicate the mechanisms of IgE overproduction in local polyp tissue. Nevertheless, the mechanisms that influence the local differentiation and function of B cells and antibody production remain unclear, and the pathways for the local activation of B cells are still controversial.

### Local immunoglobulin production is enhanced in NPs

The activation of IgM- and IgD-producing B cells is followed by the generation of IgG-, IgE-, and IgA-producing B cells *via* class switching (**Table 1**). The levels of IgG, IgG subclasses(IgG1, IgG2, IgG3, and IgG4), IgM, IgE, and IgA were significantly increased in polyps compared with CRSsNP and controls samples, and showed little correlation with serum Ig levels (13). In addition, the levels of IgG, IgG4, and IgE were significantly increased in SAE IgE(+) NPs compared with SAE IgE(-) NP patients, while the levels of IgG2 were decreased in the SAE IgE(+) group (13). The number of IgM^+^, IgD^+^, and IgA^+^- plasma cells in tissue and the levels of IgM, IgD, IgA in nasal secretions were also significantly higher in CRSwNP than in CRSsNP, as confirmed by others (32–35). Also, significantly elevated IgE and IgG4 levels were observed in nasal polyp tissue from aspirin-exacerbated respiratory disease patients (36).

**Table 1:** The levels of Ig classes in NPs compared with control.

	IgM		IgD			IgG						IgE	IgA	
Phenotype	tissue	nasal secretion	tissue	serum	nasal secretion	tissue	nasal secretion	IgG_1_	IgG_2_	IgG_3_	IgG_4_	tissue	tissue	nasal secretion
								tissue	tissue	tissue	tissue			
**CRSsNP**	**—^13^**	**—^37^**	**↑^43^↑^37^↑^38^**	**—^43.38^**	**—^37^**	**—^13.40^**	**—^13^**	**—^13^**	**—^13^**	**—^13^**	**—^13^**	**—^13^**	**—^13.37.39.40^**	**—^39^**
**CRSwNP**	**—^13^↑^37^**	**↑^37^**	**↑^37^↑^43^**	**—^38^**	**↑^37^**	**↑^13.40^**	**—^37^**	**↑^13^**	**↑^13^**	**↑^13^**	**↑^13.42^**	**↑^13^**	**↑^13.37.39.40^**	**↓^39^**
**non-eos NP**	**—^10^**		**↑^43^—^37^**	**↑^43^**		**↑^10^**					**↑^42^**	**—^10^**	**↑^10^**	
**eos NP**	**—^10^**		**↑^43^**	**↑^43^**		**↑^10^**					**↑^41^**	**↑^10^**	**↑^10^**	

1. ↑: high Vs control subjects(healthy sinus mucosa);

2. ↓: low Vs control subjects(healthy sinus mucosa);

3. —: no significant differences Vs control subjects(healthy sinus mucosa);

4. The numbers in the table are the number of the reference from which the data is taken;

5. CRSsP, CRS without (sine) polyps; CRSwP, CRS with polyps; Ig, Immunoglobulin; non-eos NP, non-eosinophil nasal polyp; eos NP, eosinophil nasal polyps.

Classically, among these Ig isotypes, IgG4 and IgA can compete with IgE for binding to allergens, thus preventing the formation of allergen/IgE/receptor complexes. In NPs, recent studies also showed that locally elevated IgG could antagonize IgE-mediated proallergic inflammation (19, 37). Meanwhile, IgD-activated mast cells and PD-1^high^CXCR5^-^CD4^+^ T cells were found to participate in local immunoglobulin production independent of ectopic lymphoid tissues in patients with CRSwNP (38, 39). IL-5/Il-5α signaling was reported to enhance local B cell differentiation, proliferation, and survival, which could lead to increased generation of antibodies within the inflamed polyp tissue[41]. All these confirm the presence of locally generated IgE and locally activated B cells, although the complex mechanisms are not clear.

### Local class switching to IgE is up-regulated in NPs

There is ample evidence that up-regulated class switching resulting in IgE production occurs in NPs. Local IgE levels and class switching to IgE were increased in polyp tissue compared with AR and controls tissues (16). Investigation of switch circle transcripts revealed ongoing local CSR to IgE, as evidenced by enhanced expression of key markers of the process, including IL-4, ϵ-germline gene transcripts(ϵ-GLT), ϵ-mRNA, and IgE protein. Increased local class switching to IgE and production of IgE were (17) also demonstrated to be significantly higher in terms of mRNA expression for ϵ-GLT, and enzyme activation-induced cytidine deaminase(AID) in NPs than in control tissues. In addition, the elevated expression of molecules required for Ig production, the generation of T follicular helper(Tfh) cells, and the production of IgE in eosinophilic polyps were shown (14). Furthermore, upon *in vitro* stimulation with Dermatophagoides pteronyssinus 1(Der p 1), a specific circle transcript of Iϵ-Cm (408 bp) produced during CSR from IgM to IgE was detected to be increased in eosinophilic NPs, meaning Ig class switching to IgE in eosinophilic polyps with ectopic lymphoid tissues (10). This evidence confirms the occurrence of local class switching to IgE in polyp tissue.

## Modulation of local IgE production

### Microbial colonization stimulates local IgE formation, especially SA

In nasal mucosa, local IgE production can be triggered not only by allergens (7, 40) but also by microbial pathogens, such as bacteria (40) and rhinovirus (41). Significantly higher levels of IgE antibodies specific for nasal bacteria, including *Staphylococcus aureus*(SA), *Staphylococcus pyogenes*, and *Haemophilus influenzae*, were found in polyps of patients with CRSwNP (42). Among these microbial pathogens, SA is one of the most important factors stimulating IgE formation in NPs. SA is a gram-positive bacterium and common human colonizer, and given the opportunity, it can become a dangerous pathogen (43, 44). Patients with NPs have been shown to have higher rates of SA colonization (63.6%) than those with CRSsNP(27.3%) and healthy adults(33%). Moreover, in the subgroup of NP with asthma and aspirin intolerance, SA colonization was present in 87% (45–47).

On the one hand, SA can act as an allergen and induce the formation of IgE with specificity for SEs in polyps, which is independent of serum total and specific IgE (12). Local SE-IgE has been demonstrated to rapidly degranulate mast cells and basophils upon contact with the specific antigen and to facilitate antigen binding to B cells (8, 19). Local SE-IgE is predictive of concomitant asthma and associated with severe local eosinophilic inflammation, suggesting the potential role of SE as a disease modifier (48). On the other hand, SA can release enterotoxins(i.e., SEs) that act as superantigens and effectively modify the functions of T and B cells, eosinophils, and other inflammatory as well as structural cells. Superantigens directly activate T cells by crosslinking the Vβ chain of T-cells to major histocompatibility complex class II on antigen-presenting cells(polyclonal activation), which is different from the T-cell activation induced by classical peptide antigens (46). Superantigens stimulate proliferation and effector function in Th2 cells, induce the release of IL-4 and IL-13, and stimulate class switching to IgE and finally IgE formation in CRSwNP (49). This activity is nonspecific and polyclonal and leads to massive inflammatory mediators release, resulting in inflammation. Meanwhile, staphylococcal protein A(SpA) produced by SA, can also act as a B-cell superantigen and induce multiclonal B cell activation, which also triggers the production of polyclonal IgE by plasma cells (49). Not only in nasal polyps, but also in asthma, specific IgE to SE-IgE can frequently be detected in serum and has been associated with severe asthma defined by hospitalizations, oral steroid use, and a decrease in lung function. Even serum SE-IgE was demonstrated to predict the development into severe asthma with exacerbations over the next decade.

### Dysfunction and dysregulation of B cells affect antibody synthesis

In healthy nasal mucosa, B cells and their derived plasma cells are relatively rare (30), while expansion and activation of B cells occur in the nasal mucosa in patients with CRSwNP, which results in the overproduction of antibodies and local inflammation. Several recent reports have shown an immunodeficiency and specific antibody deficiency in CRS (50–53), suggesting that dysfunction and dysregulation of B cells play a pathogenic role in CRS. However, the mechanisms are still unclear.

B cell dysfunction is partially associated with genetic defects. CD19 functions with both, CD21 and CD81, to enhance signaling from the B cell receptor. Mutations in these receptors are associated with hypogammaglobulinemia, decreased memory B cells, and impaired specific antibody responses (54, 55). In addition, mutations in the receptors for B-cell activating factor (BAFF), BAFF receptor(BAFF-R) and transmembrane activator and calcium modulator and cyclophilin ligand interactor(TACI) lead to hypogammaglobulinemia (56–60) and TACI mutations are also related to sIgA deficiency (59, 60). B cell functions triggered by the B cell receptor are regulated by several germline-encoded receptors, including Toll-like receptors(TLRs) and complement receptors(CRs). In pathological conditions, the role of B cell-expressed TLRs and CRs is important in B cell dysfunction. For example, SA triggers B cell activation *via* SpA-induced sensitization of B cells to TLR2-active lipopeptides. Combined SpA- and TLR2-mediated B cell activation promotes B cell proliferation (61). The excessive activation of B cells *via* TLRs or CRs is associated with several diseases, including sepsis, B cell malignancies, asthma, and autoimmunity. In addition, autophagy and mitochondria also play roles in B cell development, activation, and differentiation (62).

In CRSwNP, factors promoting the activation of B cells include inflammatory cytokines [i.e., IL-5 (36), IL-6, BAFF, and APRIL(a proliferation-inducing ligand)], chemokines [C-X-C motif ligand 12(CXCL12), C-X-C motif ligand 13(CXCL13)], and complement pathway products(C3d) (63, 64). Our group previously described a significantly elevated terminal complement complex (C5b-9), and anaphylatoxins (C3a, C5a) are present in nasal polyp tissues (65). Studies demonstrated that Type-2 innate lymphoid cells(ILC2s) directly induced EBI2 expression on B cells and that some B cells might be activated *via* an extrafollicular environment, which is distinct from classic GC-mediated mechanisms (18). Recently, new strategies for B cell modulation were reported in autoimmune diseases. These therapies, such as anti-CD19 mAbs(inebilizumab, blinatumomab), anti-CD20 mAbs(rituximab, ofatumumab, ocrelizumab), and anti-CD22 mAbs(Epratuzumab), target B cell surface markers (CD19, CD20, CD22), activating factors(BAFF, TACI), or cytokines(IL-6, TNFα, IFNα) to modulate the function of B cells, which may open new therapeutic avenues to regulate B cell activation for the treatment of CRSwNPs (66–68).

### CSR to IgE is regulated by lymphocytes, cytokines, transcription factors, and B cell-intrinsic factor

T cells and the related cytokines and numbers of other transcription factors play a major role in CSR (**Table 2**). Both Tfh and Th2-like cells are helper cells for B cell class switching to IgE, while IL-10^+^, IL-35^+^, and TGF-β^+^ inducible Treg(iTreg) cells can antagonize ϵCSR in activated B cells (10, 89, 90). In addition, ILC2s may directly drive B cell activation and class switching to IgE in an antigen-independent manner (91, 92). ILC2s produce type 2 cytokines to promote type 2 inflammation upon stimulation with epithelium-derived cytokines, such as IL-25, IL-33, and thymic stromal lymphopoietin(TSLP) (1). IL-33 also activates dendritic cells(DCs), which may prime naive T cells to develop into Th2 cells *via* superantigens (93, 94). The Th2 cytokines IL-4 and IL-13 direct class switching to IgE, whereas IFN-γ, TGF-β, IL-2, and IL-21 inhibit class switching to IgE (69–72). TGF-β induces class switching to IgA and IL-21 promotes the production of IgG1. In T cell-dependent class switching, T cell-derived cytokines regulate switching and direct T and B cell collaboration and normal class switching through CD40. However, in T cell-independent class switching, BCR signaling in concert with TLR signaling can induce class switching in the absence of T cell help in response to microbial pathogens, allergens, and vaccines (95).

**Table 2:** Factors involved in the regulation of CSR to IgE .

Type	Modification on CSR to IgE	Effect on IgE synthesis	ref
lymphocyte			
Tfh, Th2-like cells	direct class switching to IgE	**(+)**	(10)
IL-10+	antagonize ϵCSR in activated B cells	**(-)**	(69)
TGF-β+-iTreg	antagonize ϵCSR in activated B cells	**(-)**	(69)
IL-35+	antagonize ϵCSR in activated B cells	**(-)**	(70)
ILC2	B cell activation and class switch to IgE in an antigen-independent manner	**(+)**	(71, 72)
**cytokine**			
IL-4	direct class switching to IgE	**(+)**	(73, 74)
IL-13	direct class switching to IgE	**(+)**	(74)
IFN-γ	inhibit class switching to IgE	**(-)**	(73)
IL-21	inhibit class switching to IgE	**(-)**	(75)
TGF-β	inhibit class switching to IgE	**(-)**	(76)
IL-2	inhibit class switching to IgE	**(-)**	(76)
IL-25	epithelium-derived cytokines, promote type 2 inflammation and class switching to IgE	**(+)**	(19)
IL-33	epithelium-derived cytokines, promote type 2 inflammation and class switching to IgE	**(+)**	(19)
TSLP	epithelium-derived cytokines, promote type 2 inflammation and class switching to IgE	**(+)**	(19)
**transcription factor**			
E2A	induce early development and commitment to the B-cell lineage	**(+)**	(77)
NF-κB	induce expression of BLIMP1	**(+)**	(78)
PU.1	induce early development and commitment to the B-cell lineage	**(+)**	(79)
PAX5	induce early development and commitment to the B-cell lineage	**(+)**	(80)
AP-1	synergize with pSTAT6 to activate germline Iϵ transcription	**(+)**	(81)
C/EBP	synergize with pSTAT6 to activate germline Iϵ transcription	**(+)**	(81)
SWAP-70	regulate antagonistic STAT6 and BCL6 occupancy of the Iϵ promoter	**(+)**	(82)
Id2	suppresses B-cell-specific gene transcription, block transcription of Cϵ GLTs	**(-)**	(77)
BCL6	inhibit IL-4-mediated CSR to IgE by blocking activation of STAT6	**(-)**	(83)
CD45	inhibit IL-4-mediated CSR to IgE by blocking activation of STAT6	**(-)**	(84)
**B cell intrinsic factor**			
AID	initiates the CSR process through DNA deaminase activity	**(+)**	(14)
M1 primer	an extracellular 52-amino acid segment in membrane IgE, induce CSR to ϵ	**(+)**	(85)
BLIMP1	induce the differentiation of IgE B cells into plasma cells	**(+)**	(86)
GFP	a carboxy-terminal 17-amino-acid-long extension of 2A peptide in membrane IgE, induce CSR to ϵ	**(+)**	(87)
**TNFRSF member**			
CD40L(TNFSF5)	enhance both germline Cϵ transcription and AID transcription through NF-κB and STAT6	**(+)**	(88)
OX40L(TNFSF4)	induce CSR on B cells	**(+)**	(88)
BAFF(TNFSF13B)	induce CSR through TACI	**(+)**	(88)
APRIL(TNFSF13)	induce CSR through TACI	**(+)**	(88)

Charcot-Leyden crystals (CLCs), composed of galectin-10 (Gal10), are proteins produced by eosinophils, basophils, and some T cells (96). They are typically found in the airways of patients with tenacious mucus, high tissue eosinophil counts, and high serum IgE concentrations (96). Recent research found that CLCs were closely related to IgE production in airway inflammation (73). The administration of CLCs with house dust mites(HDMs) boosted IgE synthesis in a mouse model compared with exposure to the presence of HDMs alone (73). In addition, crystal-dissolving antibodies suppressed airway inflammation, goblet cell metaplasia, bronchial hyperreactivity, and IgE synthesis induced by CLC and HDM inhalation in the model (73). Recent genome-wide genetic and epigenetic association studies also found a strong association of total IgE levels with hypomethylation at the CLC and LGALS10 gene loci, suggesting that crystalline Gal10 may also be a trigger for IgE synthesis in human type 2 immune disease (74, 75).

The binding of phosphorylated signal transducer and activator of transcription 6(pSTAT6) to the Iϵ promoter is essential for promoter activation. Transcription factors including E2A, NF-κB, PU.1, Pax5, AP-1, C/EBP and SWAP-70 synergizes with pSTAT6 to activate germline Iϵ transcription (76, 97–101). In contrast, the transcriptional inhibitor B cell lymphoma 6(BCL6) (77) and the transmembrane phosphatase CD45 can inhibit IL-4-mediated CSR to IgE in human B cells (78) by blocking the activation of signal transducer and activator of transcription 6(STAT6). In addition, the transcription factor inhibitor of DNA binding 2(Id2), induced downstream of TGF-β signaling, is also a negative regulator of the Iϵ promoter (76).

In studies focusing on B cell-intrinsic factors, three different IgE reporter mice were used to explore the regulation of IgE class switching and production. With M1 prime GFP knock-in IgE reporter mice, Talay O described that M1, an extracellular 52-amino-acid segment present in membrane IgE, induced CSR to ϵ (79). With Verigem IgE reporter mice, Yang Z described a predisposition for IgE-expressing germinal center B cells to differentiate into short-lived IgE plasma cells. In addition, B lymphocyte-induced maturation protein 1(BLIMP1), a plasma cell differentiation factor, was expressed at significantly higher levels in IgE B cells than in B cells producing other antibody types and inducing the differentiation of IgE B cells into plasma cells (80). With CϵGFP IgE reporter mice, He J. S reported that a GFP-tagged carboxy-terminal 17-amino-acid-long extension of the 2A peptide expressed in membrane IgE induced CSR to ϵ. Moreover, IgE plasma cells and IgE memory responses primarily arose from sequential class switching through an IgG1 B cell stage (81).

In addition, tumor necrosis factor receptor superfamily(TNFRSF) members play key roles in B cell development, maturation, homeostasis, activation, and differentiation, also influencing the ability of B cells to synthesize IgE antibodies and act as regulators of immune responses, such as CD40(TNFRSF5) and OX40L(TNFSF4), BAFF(TNFSF13B) and APRIL(TNFSF13) (82). BAFF, and the related cytokine APRIL, are important for B cell survival, proliferation, and T cell-independent class switch recombination.

In nasal mucosa, the production of IL-4 and IL-13 creates the possibility for class switching of B cells to IgE-positive B cells, and the maturation of B cells into IgE-producing plasma cells (83, 84). Blocking IL-4 and IL-13 attenuated the expression of ϵ RNA in nasal mucosa from AR patients, which indicated that allergen– dependent induction of ϵ GLT was mediated by locally produced IL-4 and IL-13 (85). In NPs, elevated levels of IL-4 and ϵ-GLT, ϵ-mRNA, and local IgE were detected, implying local CSR to IgE (16). Meanwhile, overproduction of BAFF in NPs has been demonstrated, and the subepithelial expression of BAFF was associated with increased numbers of B cells and plasma cells and increased production of IgA in patients with CRSwNP. These results suggest that BAFF might contribute to the pathogenesis of CRSwNPs *via* local induction of IgA (86, 87).

### The relationship between IgE and atopy in NP is still unclear

Allergy has been recognized as a cause of NPs since the early 1930s (88). Research showed that 51-86% of patients with NP were sensitized to at least one aeroallergen (102, 103), and sinus disease could worsen during the allergen season (104). However, this suggestion was challenged later. A retrospective study by Settipane and Chafee (105) demonstrated that more NPs were found in the nonatopic group than in the atopic group. Subsequent studies demonstrated that multiple positive skin test responses were less common in patients with NPs than in the general population (3). In addition, elevated total serum IgE was observed in aspirin-exacerbated respiratory disease, even though atopy was not present (106).

As we all know, IgE is a key player in allergic airway diseases, such as AR and allergic asthma, while the biology of IgE is not limited to the mediation of allergic diseases. As previously described, growing evidence has indicated increased local IgE production in NPs regardless of systemic atopy and nonatopy (1–4). In atopic NP, local specific IgE production can be the effect of stimulation with allergen–HDM (107). However, local hyperimmunoglobulinaemia is also present in non-atopic patients. Higher levels of specific IgE for cockroach and plantain in NP and local antigen-specific IgE in 57% of non-atopic polyp tissue were observed (108), implying the role of localized mucosal specific IgE in NP without evidence of systemic atopy.

Thus, nasal polyposis is considered a “non-allergic” disease and local IgE is independent of atopy. Our group proposed that patients may develop sensitization and allergy early in life, and in contrast, NP is mostly a late-onset disease with a different IgE pattern. A NP patient may be skin prick test positive, independent of polyclonal IgE formation occurring later in life (109), or maybe SPT negative even in the presence of polyclonal IgE in NPs.

## The function of IgE and anti-IgE treatment in NPs

### The function of local IgE

IgE antibodies are best known for their role in inducing immediate hypersensitivity reactions. Polyclonal IgE is thought to contribute to chronic T2 inflammation in CRSwNP. Several publications have demonstrated the increased levels of IgE in NPs, and the function and specificity of local IgE have been proven. In a previous study, we showed that IgE from NP tissue homogenates mediated mast cell activation and basophil degranulation (7). Recently, Shamji MH et al. confirmed the role of local IgE antibodies in CRSwNP. Specific IgE antibodies in nasal polyp tissue promoted the CD23-mediated pro-allergic response and induced basophil activation and histamine release (19). These implicated IgE an important mediator of nasal polyposis pathology. Furthermore, the functionality of local specific SE-IgE antibodies from polyp homogenates has been proven. It promoted FcϵRI- and FcϵRII-mediated pro-allergic responses by rapidly degranulating mast cells and basophils upon contact with the specific antigen, functioning as an allergen in this situation, and facilitating antigen binding to B cells (7, 19). All these effects trigger the release of T2 cytokine(IL-4, IL-5, IL-13) and eosinophil infiltration, thus facilitating chronic T2 inflammation in NPs.

### Anti-IgE treatment in NPs

Local IgE antibodies in patients with CRSwNP appear to be functional and involved in the regulation of chronic inflammation. Thus, as the important marker of NP, IgE is regarded as an ideal target for intervention. Omalizumab, a recombinant humanized monoclonal antibody against IgE, has been in use to treat allergic asthma for more than a decade. Recently, several studies were showing the effect of Omalizumab therapy on IgE-mediated multimorbidities in allergic asthma, including AR, rhinoconjunctivitis, atopic dermatitis, allergic bronchopulmonary aspergillosis, and so on (110). Omalizumab specifically binds circulating free IgE and inhibits the binding of IgE to the high-affinity receptor, thus decreasing free IgE levels and inhibiting the binding of the antibody to effector cells, consequently preventing antigen crosslinking of receptor-bound IgE and inhibiting the release of and chronic exposure to pro-inflammatory cytokines (111). Furthermore, it was known that the expression and stability of FcϵRI receptors on the surface of basophils, mast cells, and dendritic cells were regulated by IgE levels. Therefore, Omalizumab also leads to a decrease in cell surface density of IgE receptors (112). It has been proven in 2 Phase III trials to be clinically effective and safe in SE-IgE positive, allergic, and non-allergic CRSwNP and asthma. Omalizumab, adding on to intranasal corticosteroids, was associated with significant reductions in nasal polyp and CT scores, and also improved symptoms of upper and lower airways and quality of life, irrespective of the presence of allergy, which also confirmed the importance and functionality of local IgE in nasal polyposis physiopathology (6). Nowadays, anti-IgE treatment has been approved in many countries as add-on therapy with intranasal corticosteroids for the treatment of adults with severe CRSwNP for whom therapy with intranasal corticosteroids does not provide adequate disease control. Noticeably, although Omalizumab is useful in the treatment of CRSwNP, a recent network meta-analysis and systematic review showed that anti-IgE antibodies are less effective than anti-IL-4 receptor antibodies (113, 114)

## Summary

In CRSwNP, IgE plays an important role in the pathophysiology and should be considered as the central mediator of T2 inflammation. In polyp tissue, the production and function of local IgE are characteristic. First, the concentrations of local IgE are significantly increased in NPs and show weak correlations with those in serum. Second, local IgE abs are polyclonal and functional. It can induce mast cell and basophil activation and histamine release. Finally, elevated local polyclonal IgE is correlated with increased ECP, IL-5, tissue eosinophilia, and the presence of SE-specific IgE, which are also associated with asthma comorbidity and recurrence after surgery. The synthesis of local IgE is triggered by not only allergens but also microbial pathogens, especially SA. Meanwhile, it is modulated by the activation of local B cells and CSR to IgE. These effects are partially related to genetic defects and are also regulated by T cell-related cytokines, transcription factors, and B cell- intrinsic factors. Due to the central role of IgE in the local chronic inflammation of CRSwNP, it is regarded as an ideal target for therapy and anti-IgE has been proved to be clinically successful. However, the cause and main pathway of IgE production in NPs remain unknown. The mechanisms of B cell function and regulation, which are correlated with CSR to IgE, are not elucidated. The triggers of B cell activation are still unclear, and these may be specific antigens, superantigens, or B-cell dysregulation-related inflammatory factors. Answering these questions will provide valuable information to understand local IgE in CRSwNP and possibly offer the option for new therapeutic targets.

### What do we know?

1. Local IgE production occurs continuously in CRSwNP, resulting in elevated levels of IgE in polyp tissue.
2. Local IgE is polyclonal and functional, associated with Th2-inflammation in CRSwNP.
3. Local B cells are activated to produce Ig classes and class switching to IgE in CRSwNP.
4. Local synthesis of IgE is regulated by pathogen and allergen, such as SA, also B cell function, and class switching.

### Future research needs

1. What are the characteristics of local Ig production in type 2 CRSwNP?
2. What are the origin and main pathways of local polyclonal IgE production?
3. What are the triggers and mechanisms for the activation and modulation of local B cells, which are correlated with CSR to IgE?
4. How about the possibility of monoclonal antibodies targeted against B cell surface markers, activating factors, or cytokines to become new treatment options in NP?

## Author contributions

YS and NZ wrote the review and YS, YCY and SLH revised the review. CB guided the writing of the review. All authors contributed to the article and approved the submitted version.

## Funding

This study was supported by National Natural Science Foundation of China, Grant/Award Number: 81970864; Chongqing Talents Project, Grant/Award Number: cstc2021ycjh-bgzxm0080; Chongqing medical scientific research project (Joint project of Chongqing Health Commission and Science and Technology Bureau) (2020MSXM035).

## Conflict of interest

The authors declare that the research was conducted in the absence of any commercial or financial relationships that could be construed as a potential conflict of interest.

## Publisher’s note

All claims expressed in this article are solely those of the authors and do not necessarily represent those of their affiliated organizations, or those of the publisher, the editors and the reviewers. Any product that may be evaluated in this article, or claim that may be made by its manufacturer, is not guaranteed or endorsed by the publisher.

## Glossary

**Table:**

AID	enzyme activation-induced cytidine deaminase
APRIL	a proliferation-inducing ligand
AR	allergic rhinitis
BAFF	cell activating factor
BCL6	B cell lymphoma 6
BLIMP1	B lymphocyteinduced maturation protein 1
CLC	Charcot-Leyden crystals
CR	complement receptor
CRS	chronic rhinosinusitis
CRSsP	CRS without (sine) polyps
CRSwP	CRS with polyps
CSR	class switch recombination
DC	dendritic cells
Der p 1	Dermatophagoides pteronyssinus 1
ϵ-GLT	ϵ-germline gene transcripts
EBI2	Epstein Barr Virus-induced protein 2
ECP	eosinophil cationic protein
Gal10	galectin-10
GC	germinal center
HDM	house dust mite
IgE	Immunoglobulin E
ILC2	Type-2 innate lymphoid cell
pSTAT6	phosphorylated signal transducer and activator of transcription 6
SA	Staphylococcus aureus
SE	staphylococcal enterotoxin
SpA	staphylococcal protein A
STAT6	signal transducer and activator of transcription 6
TACI	transmembrane activator and calcium modulator and cyclophilin ligand interactor
Tfh	T follicular helper
TLRs	Toll-like receptors
TNFRSF	tumor necrosis factor receptor superfamily
TSLP	thymic stromal lymphopoietin
